# DBS in the basolateral amygdala improves symptoms of autism and related self-injurious behavior: a case report and hypothesis on the pathogenesis of the disorder

**DOI:** 10.3389/fnhum.2012.00341

**Published:** 2013-01-21

**Authors:** Volker Sturm, Oliver Fricke, Christian P. Bührle, Doris Lenartz, Mohammad Maarouf, Harald Treuer, Jürgen K. Mai, Gerd Lehmkuhl

**Affiliations:** ^1^Department of Stereotactic and Functional Neurosurgery, University of CologneCologne, Germany; ^2^Department of Child and Adolescent Psychiatry, University of CologneCologne, Germany; ^3^Department of Neuroanatomy, University of DüsseldorfDüsseldorf, Germany

**Keywords:** autism, self-injurious behavior, amygdala, deep brain stimulation

## Abstract

We treated a 13-year-old boy for life-threatening self-injurious behavior (SIB) and severe Kanner's autism with deep brain stimulation (DBS) in the amygdaloid complex as well as in the supra-amygdaloid projection system. Two DBS-electrodes were placed in both structures of each hemisphere. The stimulation contacts targeted the paralaminar, the basolateral (BL), the central amygdala as well as the supra-amygdaloid projection system. DBS was applied to each of these structures, but only stimulation of the BL part proved effective in improving SIB and core symptoms of the autism spectrum in the emotional, social, and even cognitive domains over a follow up of now 24 months. These results, which have been gained for the first time in a patient, support hypotheses, according to which the amygdala may be pivotal in the pathogeneses of autism and point to the special relevance of the BL part.

## Summary

A 13-year-old boy suffering from severe Kanner's autism and life-threatening self-injurious behavior (SIB) was treated with bilateral deep brain stimulation (DBS) in the amygdaloid complex. The contacts of the DBS-electrodes were positioned in the paralaminar, the basolateral (BL), and in the central nucleus (CE) as well as in the supra-amygdaloid projection system, respectively. DBS in the BL nucleus, but not in the other target areas, resulted in substantial improvement of both SIB and autism-related symptoms. Significant improvement has been achieved in the domains of social contact, affect modulation and nocturnal sleep. In addition, rudimentary speech has been initiated. These results point to the BL as a relay station which would either modulate input and output flow determining complex behaviors or represent a crucial collection of cells which are compromised in autism by unknown factors. It is intriguing, that DBS in the BL did not only improve SIB and negative symptoms related to autism but obviously “released” social and cognitive functions which had been suppressed before. For the first time we could demonstrate in a human that:

- The amygdala has an important part in the etiopathogenesis of autism and autism-related SIB
- The BL nucleus appears to be pivotal and
- DBS in the BL amygdala was effective and did not evoke any side-effects, thus having the potential for treatment of selected patients with severe Kanner's autism and related SIB.

## Introduction

SIB is a devastating disorder with a high prevalence in individuals suffering from severe retardation. It is often associated with autism. Prevalence estimates vary considerably, depending on designs and inclusion criteria of individual studies but may account for 5–10% of the mentally retarded population and at about twice that rate among autistic persons (Borthwick-Duffy, 1994; Schroeder et al., 2001). Both, chronic severe SIB and autism are extremely difficult to treat especially, if they manifest themselves in combination. So far, the most successful therapeutic strategies have been based on behavioral techniques founded on operant principles of learning/conditioning combined with pharmacological treatment using neuroleptic drugs.

The amygdaloid complex is a prominent agglomeration of nuclei and cortical regions in the medial temporal lobe (Brockhaus, 1938; Yilmazer-Hanke, 2012). Based on histological and histochemical differences, these areas are clustered into three primary units: the BL, centromedial (Ce), and cortical (superficial) parts.

Half a century ago, it had been shown that bilateral stereotactic ablation of basal and cortico-medial amygdaloid nuclei in patients with temporal epilepsy and aggressive behavior improved aggressiveness considerably (Narabayashi et al., 1963; Narabayashi and Uno, 1966; Narabayashi, 1970, 1979; Luczywek and Mempel, 1976; Mempel et al., 1979). This demonstrated the important role of the amygdala in the pathogenesis not only of temporal epilepsy but also in emotional, especially in rage processing.

Sub-nuclei of the amygdaloid complex and especially their intrinsic GABA-ergic and glutamatergic systems have recently spurred interest in the field of autism-research (Rubenstein and Merzenich, 2003; State, 2010). Plausible hypotheses have been established, linking pathological bioelectrical activity in amygdaloid circuits with some of the most predominant features of the autistic spectrum (Bachevalier, 1994, 2006; Baron-Cohen et al., 2000; Amaral et al., 2003 as reviewed by Schumann et al., 2006; Markram et al., 2008; Markram and Markram, 2010).

Here, we present the case of a now 14-year-old boy with early-childhood autism, mental retardation, and conventionally intractable SIB whom we have successfully treated with DBS in the BL amygdala based on the mentioned pathophysiological considerations.

For the first time, we demonstrate that DBS in the latter structure has the potential to markedly reduce SIB, and also to improve features pertinent to the autistic syndrome, such as deficits in social contacts, affect-modulation and speech, fear and anxiety as well as sleep disorders.

## Background

### Patient and case history

Following a pregnancy without complications the patient was born via section due to bleedings (placenta praevia). Genetic investigations did not demonstrate any chromosomal or molecular genetic abnormalities, no sign of fragile-x-syndrome and no mutation of the MECP2-gene. Diagnostics of inborn errors of metabolism, e.g., of Lesch–Nyhan syndrome, were also inconspicuous.

The patient, at that time an 11-year-old autistic (Kanner's autism) and mentally retarded male youth with infantile cerebral palsy, was referred for treatment of SIB to the out-patient unit of the Department of Child and Adolescent Psychiatry of Cologne University Hospital. In the first years of his life occasional complex partial and generalized epileptic seizures occurred which were abolished completely by anti-epileptic medication.

The patient's SIB had already developed in his infancy at age 3 and deteriorated severely and continuously during the last two years before referral. Almost permanent restraint and around-the-clock staff supervision became necessary to prevent severe self-injury. As a result of permanent body restraint and infantile cerebral palsy (severe spastic paraparesis of the lower extremities and mild spasticity in the upper extremities) the boy had to use a wheel-chair for years, thus becoming unable to stand freely or to walk unaided.

Along with SIB, features characteristic for the autistic syndrome developed which added to compromising his condition extremely. He was unable to communicate verbally, since language development had failed. Except with his parents and his elder brother, who is also suffering from Kanner's autism, no social interactions were possible. It was not feasible to establish eye contact. He did not exhibit any exploratory behavior and suffered from progredient, eventually most severe anxiety attacks, emotional tensions, and temper tantrums. Despite adequate medication, nocturnal sleep was interrupted every 1–1.5 h by prolonged periods of screaming and SIB which worsened the social situation considerably.

The severity of the boy's symptoms, especially his inability to communicate and collaborate, precluded the application of standard tests used for assessment of symptoms of the autism spectrum (e.g., The Autism Diagnostic Observation Scale, ADOS: (Lord et al., 2000), and the Social Responsiveness Scale; SRS: Constantino and Gruber, 2005). Repeated observations of the parent–child-interaction, behavioral monitoring at the kindergarten as well as administering a specific autism-interview (ADI-R; Lord et al., 1994) confirmed the diagnosis of Kanner's autism.

Initially, the patient was treated pharmacologically with neuroleptic drugs for preventing self-injury and to permit an at least transient release from permanent restraint. At age 10, neuroleptic therapy was started with Risperidone® (maximal dose of 2 mg/day). This successfully held at bay SIB for about two years. With the boy entering puberty, aggression and SIB increased, despite the administration of the maximally tolerated dose of Risperidone®.

Since the therapy with Risperidone® obviously did not suffice, medication was changed to Topiramate®, an anti-epileptic and anti-migraine agent as well as a mood-stabilizer; maximum dose of 200 mg/day. This also failed to sufficiently reduce the self-harming behavior. Therefore, the medication schedule was abandoned again and replaced by a “third-generation” atypical neuroleptic, Aripiprazole®, which was administered at 20 mg/day and markedly reduced SIB for several weeks. Subsequently, frequent episodes and outbursts of aggressive behavior recurred, calling for additional sedative medication using benzodiazepines (Lorazepam®) and permanent restraint to lessen self-injury.

In summer 2009, when the boy was 13-years-old, life-threatening SIB could no longer be prevented or averted with psychopharmacological treatment regimens and behavioral therapy alone. At this point, neuromodulation using DBS in the amygdaloid nuclei and the supra-amygdaloid projection system was considered.

After having received informed consent by both parents as well as a permit by the ethical committee (IRB) of the University of Cologne, surgery was performed 10/23/2009 and DBS initiated. In addition to DBS, pharmacological treatment with Aripiprazole® at 20 mg/day was continued over the first year of follow-up and gradually reduced thereafter.

### Ethical implications

The parents are court-approved guardians. It goes without saying that they had been fully informed well in advance about the experimental nature of this last resort treatment, about realistic expectations and, in particular, about all conceivable risks of DBS in the amygdala and the supra-amygdaloid projection system. Since the amygdala has a low threshold for afterdischarges induced by electrical stimulation (Akert, 1961; Falconer et al., 1964), the possible risk of generating epileptic seizures by DBS had been explained explicitly.

The fact, that this nuclear complex and its projection system were proposed as targets for DBS for the first time was explained in quite some detail in repeated talks with the parents.

IRB of the Medical Faculty of the University of Cologne had been informed in considerable detail beforehand and granted consent to undertaking a so-called “Individueller Heilversuch.” This legal term roughly translates into “individual attempt at healing.” In a strict sense, under German law the consent to an “individual attempt at healing” by the ethical committee would not have been mandatory in conventionally untreatable disorders like the one discussed here. An “Individueller Heilversuch” precludes scientific evaluation. Thus, the treatment had been performed just to improve the deleterious condition of the patient. The permit to retrospectively analyze our data has been given by our ethical committee in a late stage of follow-up on our demand.

### Surgery

Targeting and trajectory planning was described elsewhere in detail (Voges et al., 2002). For geometry-correction and landmark identification, a pre-operative MRI data set was obtained with an 1.5 T Gyroscan Intera (Philips Medical Systems) scanner equipped with SENSE-flex-M surface coils (imaging parameters: T1-weighted, gradient echo, TR 31 ms, TE 15 ms, flip angle 40°, three dimensional encoding, bandwidth 35 Hz/pixel, field of view 290 mm, slice thickness 2 mm, slice distance 2 mm, matrix 512 × 512, stack of 70 slices) and was fused with CT data obtained immediately preoperatively with a stereotactic localizer attached to the patient's skull. Here, a Philips iCT 256 was used. Image geometry was as follows: slice thickness 1.25 mm, slice spacing 1.25 mm, field of view 310 mm, matrix 512 × 512. A total of 110 slices was recorded. In order to define the target-areas in more detail, we applied a non-linear transformation of the delineations of matching structures using the Atlas of the Human Brain by Mai et al. (2008).

Under general anesthesia two quadripolar DBS-electrodes (Medtronic Inc., model 3387 R) had been stereotactically implanted bilaterally through precoronal burr holes into:

1. The amygdaloid complex and
2. The supra-amygdaloid projection system including the bed nucleus of the stria terminalis, respectively.

The non-insulated alloy poles of the electrodes employed are ring-shaped with a diameter of 1.27 mm and a height of 1.5 mm. Four such contacts are stacked along the stimulation electrode in a way that they are separated from each other by insulating spacers being equally 1.5 mm high. This electrode-/contact-geometry permit's stimulating a cylindrical volume with an approximate maximum diameter of 4–5 mm and an estimated length of 10.5 mm. All four individual electrodes were fixed within their respective burr holes with sutures and bone-compatible cement. In the same session, they were subcutaneously connected to impulse generators (IPG) (Medtronic Inc., “Activa PC®”) which were bilaterally implanted in a pocket under the cutis over the breast muscle.

### Targeting

#### Amygdala-electrodes

In humans, the precise targeting of amygdaloid subnuclei is complicated by a quite marked degree of variability concerning the locations of its components within the 3D-stereotactic space as well as to their individual spatial relationships (Baird et al., 1960; Oya et al., 2009). Hence, a sufficiently precise definition of the targets of the electrode-contacts is not feasible in terms of stereotactic coordinates. Instead, individual MRI-data of each patient have to be matched with atlas templates (Löchel, 2007).

DBS targets were chosen in both hemispheres as to contact the entire dorso-ventral extension of the deep (BL) nuclei, the supra-amygdaloid region, comprising the lateral division of the CE and, finally, the sublenticular area with the CH 4 group of cholinergic cells and passing fibers.

***Left electrode; positioning of stimulation contacts***. Two contacts were placed within the BL-amygdala: a first one, in the border region between the ventral (paralaminar) and the BL nucleus (Contact 0) and a second one (Contact 1) in the center of the BL nucleus. Due to the presumably significant current spread, depending as to strength and range on the stimulation parameters used (McIntyre, 2011); the immediately adjacent parts of both the lateral and basomedial nuclei might be affected likewise.

The third Contact (Contact 2) was placed within the most lateral division of the CE.

Contact 3 was positioned in the sublenticular territory which encompasses several poorly segregated major structures: the striatopallidal amygdalofugal fibers, fibers related to the external capsule, the uncinate fascicle as well as the magnocellular cholinergic system of the forebrain and the extended amygdala (Alheid and Heimer, 1988).

**Figure 1:**
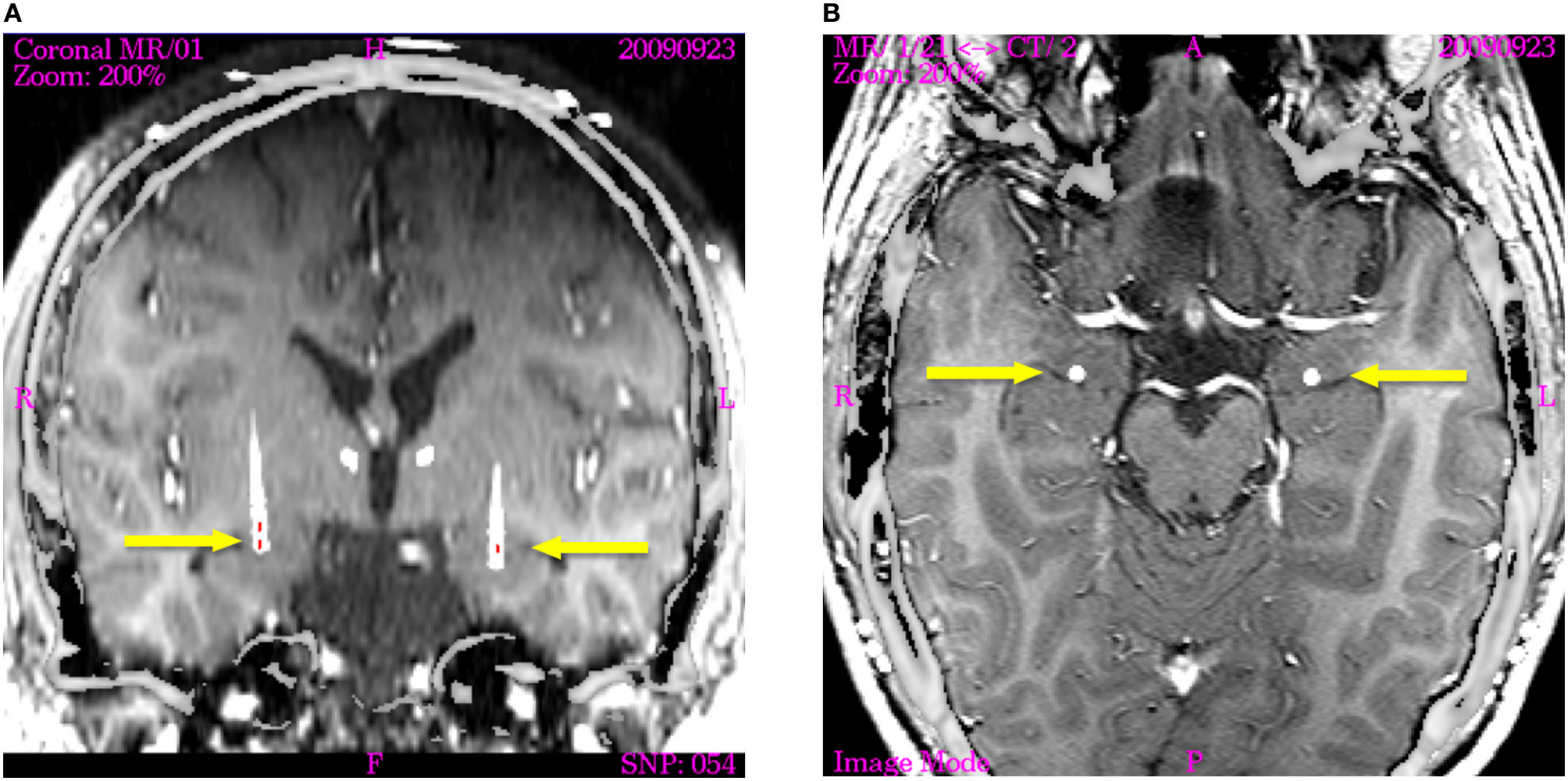
**Image fusion of post-operation CT (nine months after implantation) with T_1_-weighted intraoperative planning MRT visualizing the actual electrode position in axial and coronal planes (displayed image size: 145 × 145 mm).** The active contacts are indicated by red lines in the coronal section. **(A)** The coronal image is re-sliced in order to show the electrode trajectory in the section plane. Arrowheads indicate the trajectories of the supra-amygdaloid electrodes, which are not activated. The active poles on the right electrode are located close to the posterior part of the basolateral amygdaloid nucleus (Contact 0 and 1). The active pole on the left electrode is positioned in the center of the basolateral nucleus. **(B)** Horizontal section through the amygdala of both hemispheres showing the electrodes indicated by arrows in the posterior part of the basolateral nucleus immediately in front of the tip of the temporal horn of the ventricle.

***Right electrode; positioning of stimulation contacts***. The right electrode was positioned corresponding to the one on the left side, but 2 mm more dorsally. Thus, Contacts 0 and 1 are placed within the posterior part of the BL nucleus, Contacts 2 and 3 are located in the sublenticular fiber system (Figure 1).

#### Electrodes in the supra-amygdaloid projection system

The distal pole (Contact 0) was positioned in the fasciculosus nucleus of the thalamus (the primary target of fibers of the ventral amygdalofugal pathway and the inferior thalamic peduncle).

**Table:** 

Contact 1	targeted the rostro-dorsal border of the fasciculosus nucleus/inferior thalamic peduncle.
Contact 2	was positioned in the supra-amygdaloid fiber system, while
Contact 3	targeted the lateral part of the bed nucleus of the stria terminalis which is a major projection area of the central amygdala.

### Documentation of electrode localization

The correctness and precision of electrode placements at their respective targets had been assessed with intra-and post-operative stereotactic X-ray images, registered with the intra-operative MRI data sets, according to which targeting and trajectory planning had been performed. Nine months after implantation, the precision of the electrode placement as well as the stability of their positions have again been verified by a control CT, registered with the intra-operative planning-MRI and mapped onto the atlas of the human brain (Mai et al., 2008) Using the various data sets, we thus determined the most probable anatomic position which was unchanged when compared to the immediate post-operative control.

### Stimulation parameters, sites, and schedules

Since the boy's deleterious condition required rapid improvement and the functional relevance of the targeted substructures with respect to SIB and autism was but speculative, three days after surgery all four contacts of the amygdala—electrodes had been activated simultaneously. This and subsequent additional stimulation of the two proximal contacts of the supra-amygdaloid electrodes which targeted the extended amygdala as well as the bed nucleus of the stria terminalis, only led to insufficient improvement.

We therefore restricted DBS, by successively eliminating initially active contacts, to those targeting the BL and the Ce nucleus. It turned out that only stimulation over the contacts in the BL amygdala (Contact 1 of the left and Contacts 0 and 1 of the right amygdala-electrode) induced significant and stable improvement of both SIB and symptoms pertinent to the autism spectrum. Stimulation over the contacts targeting the CE nucleus was not only inefficient, but appeared to even worsen the condition of the patient.

Permanent DBS was performed with charge-balanced electrical square wave pulses of 120 μs duration, 130 Hz pulse following frequency and 2–6.5 Volts amplitude. Voltage was slowly increased in the course of 12 months. Choosing a constant frequency in this range has led to optimum results in the past in a robust and reproducible way in the treatment of both motor and several psychiatric disorders (Benabid et al., 1994, 2001, 2009; Nuttin et al., 1999).

## Results

### Self-injurious behavior (SIB)

The patient's parents longitudinally monitored and documented the behavior of their son in detail. The father developed an empirical score of his own design to rate the degree of SIB, as indicated by an ordinal scale from 6 (worst case) to 1 (best case), describing the extent of physical fixation required to control self-injury inflicted by auto-aggression (1: no restraint necessary; 2: restraint of the wrists suffices and is well tolerated; 3: restraint of the wrists prevents self harm in an almost adequate manner; 4: restraint of the wrists fails to completely control SIB; 5: restraint is not sufficient to control SIB—however, skin lesions do not occur; 6: restraint does not prevent skin lesions and life threatening self-injury). The score has been applied several times a week during 6 months before surgery and during the entire follow-up period.

However, this empirical score relied on subjective, day-to-day impressions of the parents who did not command medical, psychiatric, or psychological training.

As becomes clear by the nature of the observable, this general tool was made up devoid of statistical considerations, or strict attempts at standardization. Therefore, we must not take its results at face value, but have to consider biases introduced by the parents, who were in the know about changes in therapy. This also applies for the investigating child and adolescent psychiatrists and neurosurgeons. In addition, we should realize that this score is problematic when it comes to quantification, since it rests on an ordinal scale. Hence, it is rather an aid for recording observation than an instrument fit for scoring the severity of specified symptoms or behavioral patterns.

SIB and symptoms of the autism spectrum were assessed by the child and adolescent psychiatrist at irregular intervals for two years before and according to clinical necessities, at minimum at three months' intervals, after surgery. After 10 months of stimulation the battery of the amygdala-IPG was depleted and the boy was without stimulation for 4 weeks. Additional investigations have been performed during the period without stimulation, as well as 1 and 6 weeks, respectively, after exchanging the exhausted IPG that supplied the amygdala-electrodes and resuming of DBS and finally on January 24th, 2012.

Any of the pharmacological treatment schedules outlined above were, at best, beneficial to improve SIB up to scores lower than 6 but not lower than 4. In the final pre-operative phase of the disorder, scores were constantly 6 for more than 8 weeks.

DBS in the amygdala resulted in a gradual, but very unstable improvement of SIB over the first 10 months after surgery to levels lower than 3. During this period, the boy was—on occasion—able to control his behavior for several hours usually followed by severe attacks of SIB. In the 11th post-operative month DBS was interrupted for 4 weeks because of depletion of the impulse generator's (IPG) battery.

The father rated his son's behavior—before battery depletion—with scores of 2–3. However, these scores continuously rose to 6 during the period without stimulation and dropped to 2 one week and to 1–2 (no restraint required) six weeks after re-starting DBS. At that time, DBS was restricted to the BL nucleus. This score has been maintained since then. Nevertheless, there still have been infrequent, less severe outbursts of SIB which rarely would last longer than up to 1.5 h and usually have been triggered by unexpected changes in the patient's environment, common colds, etc.

The child and adolescent psychiatrist applied the Clinical Global Impression (CGI)-Severity Scale (Guy, 1976) which is specifically focused on target symptoms of irritability (aggression, self-injury). The behavior was rated with a score of 6 (severely ill) before surgery as well as without neurostimulation, i. e., when the battery of the implanted stimulation device had failed due to depletion, and with a score of 4 (moderately ill) one week and six weeks, respectively, after DBS had been resumed following battery replacement. The behavior remained stable with a score of 4 during the subsequent 12 months until a final rating was done on January, 24th, 2012, when the child and adolescent psychiatrist visited the family at home for a behavioral observation (ADI-R and CGI). The complete assessment of the degree of irritability vs. time is shown in Display 1.

**Figure A1:**
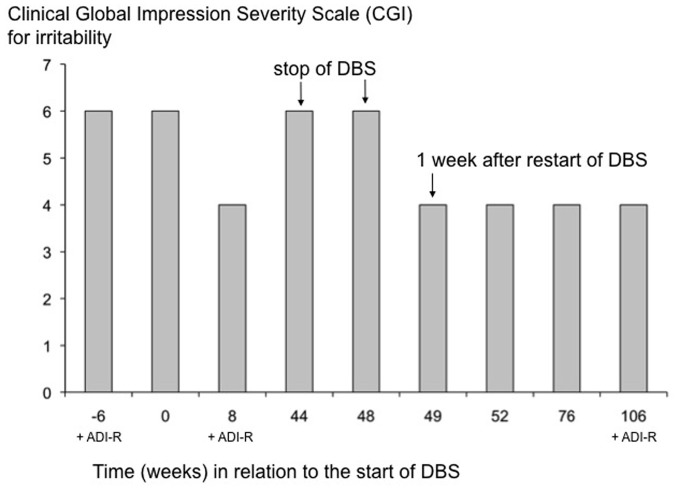
**Changes of irritability [Clinical Global Impression Severity Scale (CGI)] and of core symptoms of autism (ADI-R) in the course of treatment.** The CGI-Severity Scale according to Guy (1976) is displayed here for addressing the target symptoms of irritability expressed by the patient during the course of treatment. The scale has a range from 1 (normal) to 7 (extremely ill). Time point 0 corresponds to the initial onset of DBS. Arrows indicate substantial changes induced by stopping (week 44) and restarting (week 48) DBS, respectively. After resuming DBS, CGI was constantly rated at 4 (moderately ill). The CGI was measured for the last time 59 weeks after restarting DBS treatment. CGI was determined on the same day when the ADI-R was also assessed. ADI-R is included into this diagram for illustrative purposes.

Having exchanged the IPG and resumed DBS, it took one week until the CGI improved to a level of 4. This period was interrupted by violent fluctuations of the patient's condition. These fluctuations were reduced after the supra-amygdaloid contacts targeting the supra-amygdaloid fiber system—as well as the bed nucleus of the stria terminalis—had been inactivated, resulting in a stabilized and continuing beneficial action of therapy (Figure A1).

One week after resuming of DBS the (CE)-contacts had been inactivated. Thereafter, the clinical state of the boy improved further. This means that, according to the paternal rating, scores of 2 and even 1 were attained more frequently and remained stable for longer periods. Nevertheless, the CGI did not reach levels below four, even with exclusive stimulation of the BL nucleus of the amygdala.

## Features of autism

The ADI-R, a structured diagnostic interview suite (Lord et al., 1994; Rutter et al., 2006), was used for assessing the boy's behavior on admission to the Department of Child and Adolescent Psychiatry: Social Skills: 20; Non-verbal communication: 8; Restricted Repetitive and Stereotyped Behavior: 18. Preoperatively, ADI-R—with respect to all scales—was also over the threshold retrospectively assessed for the age of 4.0–5.0 years. This tool was re-applied post-operatively in January 2012, i.e., 57 weeks after the restart of DBS. When ADI-R was actually measured at that time, the following post-operative values were obtained (retrospective assessment at the age of 4.0–5.0 years are given in parentheses): Social Skills: 20 (20); Non-verbal Communication: 7 (8); Restricted Repetitive and Stereotyped Behavior: 13 (18). The most notable improvement was found in Restricted Repetitive and Stereotyped Behavior. This improvement was largely in concordance with the results of CGI that indicated a decrease of irritability (aggressive behavior/self-injury) from 6 (in the absence of DBS) to 4 (during DBS).

The child and adolescent psychiatrist noted an improvement in self-regulatory skills in response to visual and auditory stimuli and in the communicative eye contact with the examiner 6 weeks after resuming neurostimulation. He based his report about the behavior of the patient on the criteria of the “Autism Diagnostic Interview-Revised” (ADI-R, Lord et al., 1994).

Already at that time, the patient's parents reported that his anxious behavior resulting from social contacts as well his emotional tensions were reduced to a remarkable degree. Sleep was normalized. The boy became able to participate in activities which had been impossible before operation. He started venturing out with his father, enjoying e.g., car rides. For instance, he became able to actively explore and to play an electronic piano for up to 30 min. The parents also reported a remarkable improvement of their difficulties to aliment their son. Before the period of neurostimulation, the boy had avoided to explore new food and accepted only a very limited repertoire of different edibles. After restarting neurostimulation and restricting DBS to the (BL) the parents observed that their son found pleasure in exploring new and unknown food.

Surprisingly, after 6 months of stimulation, the parents reported the utterance of single words such as “Papa” (daddy), “Mama” (mom), and “Hunger” (hungry) in the proper context. Occasionally, when listening to pop music, he would join the singer, pronouncing syllables of the refrain. The utterance of words or comparable elements of language had been impossible previous to the stereotactic procedure.

It was also observed by the parents and the child and adolescent psychiatrist that the patient improved his skills to modulate his affects and moods after restarting neurostimulation and restriction of DBS to the (BL). The abrupt shift of balanced moods into aggressive behavioral traits was mitigated and replaced by a more adapted behavior to social situations. This improvement was documented in the behavioral observation of social interaction by the child and adolescent psychiatrist. Improvement was slow and interrupted by frequent recurrences of the symptoms. It became significant and stable after restriction of DBS to the BL nucleus by December, 2010. Since then this status has remained unchanged with a continuing tendency to further improvement. In February 2011, medication with Aripiprazole® could be reduced from 20 to 10 mg/day and Lorazepam® application even be discontinued.

## Discussion

DBS in the amygdala and its projection system has been used to treat severe, life-threatening SIB in a 13-year-old boy, suffering from Kanner's autism and mental retardation.

During a follow-up period of 26 months, not only SIB, but also core symptoms of the autistic spectrum (fear and anxiety, emotional tensions, sleep disorder, impaired self-regulatory processes in response to auditory and visual stimuli, impaired affect modulation, and impaired communicative eye-contact) improved gradually and have now reached their lowest levels. Language development, not having occurred previously, was initiated or disinhibited.

DBS in the supra-amygdaloid projection system, as well as in the central and the paralaminar amygdaloid nucleus had been ineffective, whereas restriction of the stimulation to the BL nucleus yielded the described improvements.

We chose the amygdala and its projection system as target areas because of the well-established role of this complex in rage processing (Fernandez de Molina and Hunsperger, 1959; Narabayashi et al., 1963; Narabayashi and Uno, 1966; Narabayashi, 1970, 1979; Luczywek and Mempel, 1976; Mempel et al., 1979) as well as in fear (LeDoux, 2003)—and social processing, impairment of which is believed to be central to autism (Baron-Cohen et al., 2000; Amaral et al., 2003; Schumann et al., 2006; Santos et al., 2011), and in relevance detection (Sander et al., 2003).

Before discussing our results we want to mention some caveats which should be considered in the face of the favorable outcome: as outlined in “Ethical implications” the treatment had to be performed as “Individueller Heilversuch” (individual attempt at healing) the purpose of which is just improvement of an otherwise untreatable clinical condition and not scientific evaluation. The permit to retrospectively analyze and publish our data has been obtained from our IRB on our demand in a late stage of follow-up.

Summing this up, no strict scientific investigational design could be established beforehand, since all psychiatric investigations and assessments had to be scheduled according to clinical requirements, e.g., in cases, when SIB worsened or a selected stimulation paradigm failed to improve a condition insufferable for the patient. It is evident that the gravity of the patient's illness, making communication impossible, precluded the application of standard tests for assessing the severity of symptoms of both autism spectrum and SIB. Therefore, the parental ratings and judgments which we considered very trustworthy, had also to be accounted for. Hence, the only way to clearly specify and communicate the behavioral abnormalities encountered and their change in the course of DBS treatment consisted in a thorough and detailed verbal description of our observations.

Although what we denominated “positive effects” or “symptomatic improvements” could not be rated by use of psychological standard tests like the ADOS or ADI, we consider the descriptions given here sufficiently reliable, since they were procured by highly experienced Child and Adolescent Psychiatrists and additionally incorporated the important observations of the parents in the patient's domestic setting.

In this context, the rather difficult situation arising from the fact that the patient could not be transferred to the hospital for follow-up in the first year post-OP is of special importance. After restricting the stimulation to the BL-Amygdala, the boy's condition improved in such an unexpected way that we considered to publish these retrospectively analyzed data with the consent of our local ethical committee.

Despite the obvious shortcomings in the presentation of our results being due to the circumstances described above, there can be no doubt about the major improvements not only of SIB but also of core symptoms of the autism spectrum caused by DBS in the BL-Amygdala which, according to our point of view, do merit publication, especially since this is the first attempt at treating SIB associated with Kanner's autism with DBS in the Amygdala.

However, it should be noted that both investigating physicians and observing parents could not be blinded to the treatment. Biases cannot be excluded, but knowing the rather critical attitude of the parents seems to us quite improbable.

## Stimulation parameters

The amygdaloid complex has not been addressed with DBS in humans before. For the first attempt ever to do this, we deliberately used a simple stimulus protocol for the following reasons:

- There is as yet no standard procedure for establishing an optimum parameter set for DBS stimulation of any deep brain target.
- In addition, there is consensus that no current theory is able to account for even the basic aspects of the mode of operation of electrical extracellular neurostimulation viz. neuromodulation, using multi-pole electrodes like those utilized in DBS in humans.

With the abundance of unknown factors causative for autism/SIB combined with a host of free parameters available for setting neurostimulation, simple reasoning dictated using standard settings proven to be efficient in ameliorating movement disorders (Benabid et al., 1994, 2001, 2009) as a first approximation for subsequently identifying stimulus protocols specifically aimed at obtaining favorable clinical outcomes with DBS treatment of autism/SIB.

In this context, it should be noted that we raised stimulus voltage very cautiously, since it is well known that the amygdala has a high propensity for LTP kindling (Racine, 1978) and a very low threshold for generating repetitive discharges (Akert, 1961; Falconer et al., 1964).

A frequently occurring, albeit transient clinical improvement caused by local micro-lesions following the insertion into and final positioning of DBS electrodes within other targets such as the subthalamic nucleus did not occur in our case. This phenomenon mimics actual electrostimulation at high frequencies for a couple of days despite the electrode has not been activated yet, i.e., with the neurostimulator still in the “off” mode. Therefore, its absence strongly speaks against the assumption that the therapeutic effects had been caused by micro-lesions in the case described here.

## Stimulation sites

In view of these points, the amygdala may be considered a key structure in the etiopathogenesis of autistic symptoms and in mediating aggressive behavior. However, neither ablational surgery nor functional imaging have sufficient spatial resolution for correlating their results with the subnuclear organization of the amygdala and associated areas. This makes it difficult to predict the consequences of stimulation of amygdaloid subnuclei with respect to SIB and symptoms associated with autism.

In order to assess the specific amygdaloid areas stimulation of which was likely to improve the boy's symptoms, we implanted the DBS electrodes bilaterally at two different sites. One electrode targeted the paralaminar, the BL as well as the Ce nucleus in order to modulate the input (L-BL) and successive processing area (BL–Ce) of the amygdala. The trajectory of the other electrode was destined to contact the extended amygdala in order to interfere with efferent and afferent connections.

It turned out that only stimulation within the BL amygdala improved the symptoms sufficiently, whereas stimulation of any other target yielded minor and temporarily limited effects at best. This finding strongly supports the assumption that the area which has been stimulated effectively is functionally relevant in the context of the treated disorder and that unspecific effects due to possible mechanical irritation, microlesioning, or inflammation presumably do not account for the observed outcome.

The interpretation of the results of BL stimulation remains tentative considering the complexity of the segregated circuits connected through this structure which thus might have been affected. Moreover, stimulation may not be restricted to the BL nucleus but may also affect immediately adjacent parts of the lateral and the basomedial nucleus, respectively, as a result of current spread and stimulation of intermingling dendritic trees. These nuclei and their respective connections should therefore also be considered when evaluating the effects of stimulation.

The **lateral nucleus** is the main amygdaloid target for already pre-processed multimodal information from higher order association cortices (Pessoa and Adolphs, 2010). Subcortical afferents reach the lateral nucleus predominantly from chemosensory, visual, and auditory areas of the thalamus. Accordingly, the lateral nucleus is involved in the processing and evaluation of sensory stimuli reflecting emotional and socially pertinent content (reviewed in Davis and Whalen, 2001; LeDoux, 2003).

Most of these fibers entering the lateral nucleus are glutamatergic and terminate on principal neurons that also use glutamate as transmitter. These are under inhibitory control of different classes of GABA-ergic interneurons (Lang and Pare, 1997, 1998; Yilmazer-Hanke et al., 2007; Morozov et al., 2011). Their activity plays a predominant role in balancing excitation and inhibition within the lateral nucleus. An increase in excitatory tone resulting from reduced GABAergic signaling might thus result in a hyper-excitable state causing emotional tensions, rage, temper tantrums, fear and anxiety, as well as cognitive dysfunctions (Harkin et al., 1998; Hensch et al., 1998; Schuler et al., 2001), and—in extreme cases—epileptic seizures. These states are major characteristics comprising the autistic syndrome.

The **BL nucleus** is functionally interposed between the lateral and CE and therefore regarded as a communication channel between both nuclei (see Freese and Amaral, 2009). Besides its afferents from the lateral nucleus it has major reciprocal connections with the orbitofrontal and the anterior cingulate cortex. The concurrent processing of the input from these regions may be regarded as a gate control system, where the “danger” signal, as well as other emotionally and socially relevant signals, is evaluated in the context of conscious experiences or expectations. Its activity modulates the CE but also cortical areas that are involved in the regulation of various cognitive functions (Phelps, 2006).

Hence, information propagation from cortex and subcortex through the lateral to the BL nucleus is presumably modulated by the above mentioned interaction between glutamatergic and GABAergic chemical synaptic transmission thus being particularly relevant for autism. Genetic studies/genome scans have revealed mutations in some patients with autism- associated symptoms. These genetic changes affect the transmission of both, GABA- and glutamatergic synapses (Hussman, 2001; Jamain et al., 2002; Derwińska et al., 2009, reviewed by Rubenstein and Merzenich, 2003; State, 2010). Reduced GABAergic signaling of interneurons of the L and BL nuclei might yield a “hyperexcitability-state” of the respective nuclei as found by Markram et al. (2008) and reviewed by Markram and Markram (2010) in the rat-valproic acid-model of autism and in consequence impair both intraamygdaloid and amygdalo-cortical circuitry. This might well contribute to the pathogenesis of autism as proposed by the former authors and considering the well-established role of the amygdala in rage processing also of SIB and explain the beneficial effects of DBS in the BL nucleus which have been achieved in our patient possibly through interference of DBS at high frequencies with hyperexcitable cell ensembles in the intra-amygdaloid relay-nucleus.

Nevertheless, when discussing a clinical case, i.e., a human subject, we feel ethically obliged to duly take into account that it remains controversial, whether neuroscientific results obtained from animal experimentation in phylogenetically rather primitive species, especially in rodents with their simple brain architecture and their rudimentary neocortex, are readily transferable to apparently related conditions in the hypercomplex human brain, as critically remarked by Markram et al. (2008; Markram and Markram, 2010), although such results certainly do provide a valuable starting point for future in-depth studies.

However, at this point we would like to indicate that the reported results might not be restricted to the BL nucleus alone. It is thoroughly conceivable that these beneficial effects are also related to DBS-evoked modulations exerted by the BL nucleus on a distributed network involving not only the amygdala, but also other subcortical structures such as the striatum or other basal ganglia. There are strong indications for efficient connectivity between the striatum and the amygdala, as demonstrated by Popescu et al. (2009) as well as for links with orbitofrontal regions. Recently Le Jeune et al. (2010) have shown that DBS in the subthalamic nucleus affects limbic and associative circuits. Furthermore, it was demonstrated that such modulations were related to emotional disturbances (Péron et al., 2010). Hence, these kinds of distant modulations, encompassing large-scale network effects, might also have had an important part in successfully treating the case reported here.

The improvement of core symptoms of autism by stimulation of the BL amygdala supports hypotheses ascribing the amygdala a dominant role in the pathogenesis of autism (Baron-Cohen et al., 2000; Amaral et al., 2003 as reviewed by Bachevalier, 1994; Schumann et al., 2006; Markram et al., 2008; Markram and Markram, 2010). We cannot exclude, however, that these improvements might partly or even totally be due to psychosocial alterations, caused by reducing SIB and/or pathological anxiety through DBS.

Our finding that stimulation of other amygdaloid nuclei, especially of the CE as well as of the extended amygdala has been ineffective is surprising and cannot be explained at present.

## Concluding remarks

We could show that the beneficial effects on both SIB and symptoms of the autism spectrum were due to stimulation of the BL-nucleus of the amygdala. However, long-range network effects induced by DBS of this nucleus owing to the strong connectivity between the amygdala and other deep brain structures or orbitofrontal areas may also be assumed as being causative for the positive clinical outcome reported in this case study. Moreover, it cannot excluded that psycho-social alterations caused by the reduction of SIB and anxiety may also have contributed to the improvement of symptoms of the autism spectrum The elucidation of their significance in DBS treatment of autism/SIB will remain the objective of future study.

### Conflict of interest statement

Volker Sturm is co-founder and shareholder in a start-up company (ANM GmbH, Cologne) aiming at the development and production of innovative Neurostimulators. He was supported by Medtronic Inc., with financial support of visits to some congresses. Medtronic Inc., also supported clinical DBS studies by providing impulse generators and DBS electrodes. The other authors declare that the research was conducted in the absence of any commercial or financial relationships that could be construed as a potential conflict of interest.
